# Low grade albuminuria as a risk factor for subtypes of stroke - the HUNT Study in Norway

**DOI:** 10.1186/s12883-020-01746-9

**Published:** 2020-05-02

**Authors:** Jens W. Horn, Solfrid Romundstad, Hanne Ellekjær, Imre Janszky, Julie Horn

**Affiliations:** 1 Department of Internal Medicine, Levanger Hospital Kirkegata 2, Health Trust Nord-Trøndelag, N-7600 Levanger, Norway; 2 Department of Public Health and Nursing, Norwegian University of Science and Technology, Trondheim, Norway; 3 Department of Clinical and Molecular Medicine, Norwegian University of Science and Technology, Trondheim, Norway; 4 Stroke Unit, Department of Internal Medicine, St. Olav’s Hospital, Trondheim, Norway; 5 Department of Neuromedicine and Movement Science, Norwegian University of Science and Technology, Trondheim, Norway; 6 Department of Neurology, Medical School, University of Pécs, Pécs, Hungary; 7 Institute of Behavioural Sciences, Semmelweis University, Budapest, Hungary; 8 Department of Obstetrics and Gynecology, Levanger Hospital, Nord-Trøndelag Hospital Trust, Levanger, Norway

**Keywords:** Albuminuria, Cerebral small vessel disease, Stroke

## Abstract

**Background and purpose:**

Albuminuria is a marker for endothelial dysfunction and knowledge on its association with stroke and stroke subtypes are limited.

**Methods:**

Corresponding data from 7261 participants of the population-based HUNT2 study (1995–1997) was linked with hospital records, identified all patients registered and diagnosed with a first-time stroke. Each diagnosis was validated by reviewal of the medical record appertaining to the individual. We then applied Cox proportional hazard models to estimate the hazard ratios (HRs) for the association between albuminuria (measured as albumin-to-creatinine-ratio, ACR) and diagnosis of stroke and stroke subtypes.

**Results:**

703 (9.7%) participants developed a first ischemic stroke during a median follow-up of 15 years. Higher albuminuria was associated with a higher rate for ischemic stroke and the risk rose steadily with increasing ACR (15% increment per unit increase in ACR concentration in mg/mmol). In the fully adjusted model, the HR for all ischemic strokes was 1.56 (95% CI 1.24–1.95) for those with an ACR ≥3 mg/mmol compared to participants with an ACR < 1 mg/mmol. Overall, increasing ACR was associated with a higher risk of all ischemic stroke subtypes. This was seen to be strongest for lacunar stroke (HR 1.75, CI 1.12–2.72, *p* = 0.019), and also for stroke of undetermined etiology (HR 1.53, CI 1.11–2.11, *p* = 0.009) and those caused by atherosclerosis in the large arteries (HR 1.51, CI 0.78–2.94, *p* = 0.186) than for cardio-embolic stroke (HR 1.22, CI 0.64–2.3, *p* = 0.518).

**Conclusions:**

Albuminuria is an important risk factor, potentially already at low grade, for ischemic stroke especially for lacunar subtype. Measuring albuminuria is both cheap and readily available. This offers the opportunity to evaluate the risk for endothelial dysfunction and thus the subsequent risk for stroke and cerebral small vessel disease.

## Background

The incidence of stroke has increased worldwide due to a shift in the dynamic where ageing populations are the norm and diabetes, hypertension and unhealthy lifestyle are prevalent [1]. Despite recent improvements in stroke management, stroke still frequently has devastating complications, such as loss of speech, paresis and other disabilitating conditions [1]. Lacunar infarctions account for roughly 20–25% of first time ischemic strokes and are a main manifestation of cerebral small vessel disease (CSVD) [2–4]. CSVD is usually diagnosed by MRI imaging and has been associated with, among others, an increased risk of gait disturbance and long-term cognitive dysfunction [2]. Secondary prevention of lacunar stroke has shown poor clinical benefit [2]. Although progress has been made in understanding the pathophysiology of stroke and identification of risk factors for several stroke subtypes, including cardio-embolic stroke and stroke due to large vessel disease, knowledge on the etiology of lacunar stroke is still limited. Apart from age, both hypertension and diabetes are important risk factors, but up to 23% of patients with lacunar stroke have neither hypertension nor diabetes [2, 5]. Since the brain and the kidneys share hemodynamic and anatomical similarities, it has been suggested that changes in endothelial function in the kidneys, leading to leakage of small levels of albumin, may serve as a marker for generalized endothelial dysfunction and vascular damage of cerebral small vessels [5–8]. Furthermore, according to the strain vessel hypothesis, juxtamedullary afferent arterioles in the kidney and perforating arteries in the brain are exposed to high pressure and have to maintain a high vascular tone [9]. This common hemodynamic mechanism may link albuminuria with vascular damage of cerebral vessels [9]. Beside the strain vessel hypothesis, there are other mechanisms which might contribute to endothelial damage, such as high salt use, chronic inflammation and infection or genetic causes [10, 11].

The association between albuminuria and increased risk of cardiovascular mortality [12] and stroke [2, 7, 8, 13, 14] is supported by several previous findings. However, only a few studies have examined the prospective association between albuminuria and clinically defined stroke and stroke subtypes [14]. Furthermore, it is not clear whether there is a threshold for albuminuria leading to or increasing the risk of stroke [12]. In order to address these questions, we investigated the association between albuminuria and stroke subtypes using data from the population-based Nord-Trøndelag Health Study (HUNT).

## Methods

### Study population

The HUNT Study is a population-based, prospective cohort study that was initiated in 1984. All adult residents of Nord-Trøndelag county in Norway aged 20 years or older were invited to comprehensive health surveys [15]. The present study is based on data from HUNT2 that was conducted between 1995 and 1997. Details of the HUNT2 survey have been provided previously [16]. In brief, data was collected using blood and urine samples, clinical examinations and questionnaires. Nord-Trøndelag has a population of 127,000 inhabitants and is a fairly representative county of Norway, with the exception of those counties containing large cities. The population is ethnically homogenous (less than 3% non-Caucasian) and stable with a migration rate below 0.3% per year [16]. Our study population included participants from the Microalbuminuria Study, a sub-study of HUNT2 [17]. Among 66,140 HUNT2 participants (71% of the invited residents), [16] all those with self-reported diabetes or self-reported use of antihypertensive medication, and a random sample of 5% of the non-diabetic and non-hypertensive participants have been included in this sub-study. A total of 11,519 individuals were invited, of whom 9752 have participated and 9698 delivered the 3 urine samples as requested. We excluded participants due to self-reported urinary tract infection (*n* = 176), self-reported blood in the urine the last year (*n* = 142) and menstruation while providing urine samples (*n* = 35). Furthermore, we excluded 540 participants due to self-reported history of stroke, 3 participants with a kidney failure stage 5 and 21 participants with a missing measure of glomerular filtration rate (GFR). In addition to these, another 528 participants were also excluded on the ground of missing information on the following covariates: educational status (*n* = 164), body mass index (BMI) (*n* = 84), smoking (*n* = 238), systolic blood pressure (BP) (*n* = 10), diabetes mellitus (DM) (*n* = 30) and lipids (*n* = 2), leaving 8253 participants in the final study population (Additional Figure 1).

### Exposure assessment

At the HUNT2 field stations, the participants received information about the Microalbuminuria Study by trained staff and were provided with the following a set of 3 urine cups to collect 3 first-morning-urine samples, 3 transport tubes, an envelope for returning the urine samples, written information about the Microalbuminuria Study, written instruction on how to collect the urine samples and a questionnaire. The questionnaire contains among others, questions pertaining to urinary tract infections during the previous week, persistent hematuria in the previous year and menstruation at the time of urine collection. In addition, the research team accessed information from the general questionnaires and results from the physical examinations conducted during the original HUNT2 study.

Urine samples were examined at the Central Laboratory at Levanger Hospital, Levanger Norway, using a Hitachi 91 Autoanalyzer (Hitachi, Mito, Japan). Urine albumin and creatinine were measured by an immunoturbidimetric method (antihuman serum albumin from Dako Norway, Oslo, Norway) and the Jaffé method (Roche Diagnostics, Mannheim, Germany), respectively [18, 19].

We measured albuminuria as albumin to creatinine ratio (ACR), and used a clinical classification of ACR according to the Kidney Disease: Improving Global Outcomes (KDIGO) guidelines [20]. ACR was calculated as albumin concentration in urine in mg divided by creatinine concentration in mmol. Only those participants, who delivered 3 morning urine samples on 3 different days were included in the Microalbuminuria Study, and thus the mean ACR of the three samples was calculated. Albuminuria was categorized into 4 groups, all defined as low-grade: normal albuminuria (ACR < 1 mg/mmol), mild albuminuria (ACR 1- < 2 mg/mmol), high mild albuminuria (ACR 2- < 3 mg/mmol) and moderate albuminuria (ACR ≥ 3 mg/mmol) [21].

### Outcome assessment

Data from the Microalbuminuria Study was linked to the medical records from the two hospitals providing acute medical care in Nord-Trøndelag, those being Levanger Hospital and Namsos Hospital. We identified all patients registered with a first-ever stroke diagnosis between participation at HUNT2 and December 9th, 2017 from the patient administrative system by using International Classification of Diseases-Tenth Revision (ICD-10) codes I63 and I61. The first author, JWH, a specialist in neurology with long experience in stroke treatment, validated all first stroke diagnoses by reviewing each individual medical record. However, this validation was possible only from September 6th, 2002 when the electronic medical records became available. These records provided information on symptoms, clinical findings, medical history, results from clinical examinations, including electrocardiography, duplex images, echocardiography, computer tomography / magnet resonance imaging and arteriography.

Firstly, diagnoses of acute ischemic and intracerebral hemorrhagic stroke were established. Ischemic stroke diagnoses were then categorized according to the Trial of Org 10,172 in Acute Stroke Treatment classification system (TOAST) into the following subtypes: lacunar stroke, cardio-embolic stroke, large-artery atherosclerotic stroke, or stroke of other determined etiology or stroke of undetermined etiology [22]. Possible and probable diagnoses in each category were then collated. Information from the medical records was, especially at the start of the follow up period, not always sufficient to categorize the stroke subtypes, resulting in a higher number of strokes classified as stroke of undetermined etiology. In an inter-rater reliability analysis, two raters (JWH and HE) independently validated 20 cases for stroke and stroke subtypes using TOAST classification and Cohen’s kappa was calculated for each stroke subtype and for stroke overall [23]. We found perfect consensus for ischemic stroke overall between the two raters. For subtypes we reached a moderate agreement of 70% (kappa 0.54).

In our main analyses, we used only validated stroke diagnoses, these being from when the follow up started on September 6th, 2002. In secondary analyses, we followed study participants from the date of HUNT2 examination and used unvalidated ischemic stroke diagnoses (ICD-10 code I63 from the patient administrative system) until September 6th, 2002 and subsequent validated diagnoses until the end of follow up.

### Covariates

Information was obtained on covariates from the HUNT2 questionnaire, clinical measurements and non-fasting blood samples. From these questionnaires we extracted information pertaining to a self-reported history of stroke (to ensure that we followed individuals free of previous strokes diagnosis), their status as smokers (never, former or current smoking) and their educational status (< 10 years, 10–12 years, > 13 years of education). For the 413 participants whose history was incomplete regarding education status in HUNT2, and who had previously participated in HUNT1, we relied upon the information given during the HUNT1 study which was conducted some 10 years prior to HUNT2. For our primary analysis, we conducted a complete case analysis, excluding participants with missing data on covariates.

Trained staff at the field stations collected non-fasting blood samples and recorded measurements of body height, weight and blood pressure and collected non-fasting blood samples. Height was measured without shoes to the nearest centimeter. Weight was measured, wearing light clothes without shoes, to the nearest half a kilogram (kg) and BMI was calculated as body weight in kg divided by height in meter squared. The subjects rested for a minimum of 2 min before their BP was measured 3 times with 1-min intervals by an automatic oscillometric method (Dinamap 845XT; Criticon) in a sitting position and with cuff-size adjusted to arm circumference. For our analyses, we used the mean of the second and third systolic blood pressure measurements. Blood samples were analyzed at the Central Laboratory at Levanger Hospital on a Hitachi 911 autoanalyzer. Estimated GFR (EGFR) was calculated using the Modification of Diet in Renal Disease study equation: EGFR = 175 x (Serum Creatinine in Milligrams per Deciliter)^-1.154^ x Age ^− 0.203^ (× 0.742 for Women) [24, 25].

### Statistical analysis

Baseline characteristics were presented as mean ± standard deviation (SD) or median ± interquartile range (IQR) for continuous variables and as percentages for categorical variables.

Cox proportional hazards models were applied with age as the underlying time scale to calculate age- and multivariable adjusted hazard ratios (HRs) with 95% confidence intervals (CIs) for first ever validated stroke and for its subtypes. Participants contributed person-time from September 6th, 2002, when electronic journals were accessible for both hospitals until the date of first stroke diagnosis, date of death or emigration, or end of follow-up (December 9th, 2017), whichever occurred first. We evaluated the proportional hazards assumption by comparing -ln-ln survival curves and by performing tests on Schoenfeld residuals for each covariate. We found no violations of the proportionality assumption.

Confounders were chosen based on prior knowledge on their relation to the exposure and outcome. All analyses were adjusted for age. In Model 2, we additionally adjusted for sex, smoking, educational status, self-reported diagnosis of diabetes mellitus, BMI, EGFR, systolic blood pressure, serum triglycerides and non-high-density cholesterol (calculated by subtracting serum total cholesterol – high-density cholesterol).

In a sensitivity analysis multiple imputation with 5 imputations (mi command in STATA) were applied to examine whether missing information on education (164 cases), BMI (87 cases), smoking status (256 cases), blood pressure (12 cases) and diabetes (33 cases) could have biased our estimates.

All statistical analyses were performed with STATA 15.1 for Windows (StataCorp, Collage Station, Texas).

### Results

Baseline characteristics stratified by ACR are shown in Table 1. Compared to participants in the lowest ACR category, participants with higher ACR were older, had higher systolic and diastolic blood pressure, lower EGFR and slightly higher BMI. With increasing ACR, participants were more likely to have DM and a raised likelihood to be smokers or to report lower educational levels. (Table 1).

**Table 1:** Descriptive Characteristics of the Study Population at HUNT2 (*n* = 7261)

		ACR (mg/mmol)			
		< 1 (*n* = 4564)	1- < 2 (*n* = 1561)	2- < 3 (*n* = 402)	≥3 (*n* = 734)
Age years, median (IQR)		57.7 (47.3, 68.6)	65.5 (53.6, 72.8)	66.3 (56.7, 74.1)	68.0 (57.7, 74.5)
Female, n (%)		2500 (55)	975 (62)	211 (52)	344 (46)
Education, years, n (%)					
< 10		3616 (79)	1355 (87)	356 (89)	660 (90)
10–12		684 (15)	152 (10)	33 (8)	57 (8)
> 12		264 (6)	54 (3)	13 (3)	17 (2)
Smoking status, n (%)					
never		2100 (46)	738 (47)	172 (43)	280 (38)
former		1531 (34)	514 (33)	138 (34)	280 (38)
current		933 (20)	309 (20)	92 (23)	174 (24)
Systolic blood pressure (mmHg), mean (SD)		144.9 (21.3)	154.3 (23.9)	157.6 (23.0)	160.1 (23.4)
Systolic blood pressure > 140 mmHg, n (%)		2528 (55)	1089 (70)	299 (74)	584 (80)
Diastolic blood pressure (mmHg), mean (SD)		83.8 (11.7)	87.6 (13.2)	88.4 (13.4)	88.9 (13.5)
Diastolic blood pressure > 90 mmHg, n (%)		1245 (27)	593 (38)	162 (40)	320 (44)
BMI, (kg/m^2^), mean (SD)		27.9 (4.4)	28.3 (4.5)	28.7 (4.8)	29.0 (5.0)
EGFR (ml/min/1,73m^2^), mean (SD)		88 (56.5)	86 (21.2)	84 (20.0)	79 (21.7)
HDL (mmol/l), mean (SD)		1.4 (0.4)	1.4 (0.4)	1.3 (0.4)	1.2 (0.4)
Non-HDL Cholesterol (mmol/l), mean (SD)		4.8 (1.3)	5.0 (1.3)	5.1 (1.2)	5.2 (1.3)
Triglycerides (mmol/l), mean (SD)		1.9 (1.1)	2.1 (1.3)	2.1 (1.2)	2.5 (1.5)
Use of antihypertensive medication, n (%)					
yes		2657 (58)	1081 (69)	300 (75)	540 (74)
no		1826 (40)	427 (27)	87 (22)	168 (23)
former		77 (2)	49 (3)	14 (3)	25 (3)
missing		4	4	1	1
Diabetes, n (%)					
yes		519 (11)	260 (17)	83 (21)	219 (30)
no		4045 (89)	1301 (83)	319 (79)	515 (70)
Self-reported history of cancer, n (%)					
yes		185 (4)	90 (6)	18 (4)	56 (8)
no		4054 (89)	1334 (85)	345 (86)	579 (79)
missing		325 (7)	137 (9)	39 (10)	99 (13)
Self-reported history of ischemic heart disease, n (%)					
yes		445 (10)	212 (14)	73 (18)	130 (18)
no		4106 (90)	1348 (86)	327 (81)	600 (82)
missing		13	1	2	4
Died during follow-up, n (%)		1650 (36.1)	830 (53.2)	250 (62.2)	518 (70.5)

*ACR* Albumin to creatinine ratio; *IQR* Interquartile range; *SD* Standard deviation, *BMI* Body mass index, *HDL* High density lipoprotein; *EGFR* Estimated glomerular filtration rate

Of 7261 participants, 703 (9.7%) developed a first ischemic stroke and 71 (1%) suffered from a primary intracerebral hemorrhage during a median follow-up of 15 years.

Table 2 displays the HRs and 95% CIs for the association between ACR and all ischemic and hemorrhagic stroke (Table 2). Higher albuminuria was associated with a higher rate for all ischemic strokes and the risk for ischemic stroke increased steadily with an elevated ACR. In general, HRs were attenuated somewhat in the fully adjusted model compared to those found in the age-only adjusted models. In the fully adjusted model, the HR for all ischemic strokes was 1.56 (95% CI 1.24–1.95) for those with ACR ≥3 mg/mmol compared to participants with ACR < 1 mg/mmol. When analyzing ACR as a continuous variable, the risk for ischemic stroke increased by 15% for each unit increase of ACR concentration in mg/mmol (HR 1.15, CI (1.17–1.23, *p* < 0,001).

**Table 2:** Hazard Ratios and 95% Confidence Intervals for Ischemic and Hemorrhagic Stroke by Albuminuria Among HUNT 2 Participants, (n = 7261)

			Model 1			Model 2	
ACR, mg/mmol	Cases/Person time in years	HR	95% CI	p for trend	HR	95% CI	p for trend
**All ischemic strokes**							
< 1	386/55733	1	(ref)		1	(ref)	
1 - < 2	162/16548	1.13	(0.94–1.36)		1.06	(0.88–1.28)	
2 - < 3	49/3785	1.43	(1.06–1.93)		1.25	(0.92–1.69)	
≥ 3	106/6130	1.9	(1.53–2.36)	< 0.001	1.56	(1.24–1.95)	< 0.001
**Hemorrhagic stroke**							
< 1	29/55733	1	(ref)		1	(ref)	
1 - < 2	31/16548	2.59	(1.55–4.31)		2.58	(1.54–4.33)	
2 - < 3	4/3785	1.43	(0.5–4.07)		1.38	(0.48–3.97)	
≥ 3	7/6130	1.51	(0.66–3.45)	0.095	1.46	(0.63–3.42)	0.124

*ACR* Urine albumin-creatinine ratio; *CI* Confidence interval; *HR* Hazard ratio

Model 1 is adjusted for age

Model 2 is additionally adjusted for sex, smoking status, educational status, BMI, EGFR, DM, systolic blood pressure, non-HDL Cholesterol, Triglycerides

In contrast, we found no consistent, linear association of ACR with hemorrhagic stroke. The highest risk of hemorrhagic stroke was observed among participants with ACR 1- < 2 mg/mmol (HR = 2.58, 95% CI 1.54–4.33) when compared to those with ACR < 1 mg/mmol.

Table 3 presents the associations between ACR and different ischemic stroke subtypes. Overall, increasing ACR was linked to a higher risk of all ischemic subtypes (Table 3). The associations were stronger for lacunar stroke, for stroke of undetermined etiology and for stroke caused by the atherosclerosis in the large arteries than for cardio-embolic stroke. Compared to participants with ACR < 1 mg/mmol, the HRs for those having an ACR ≥3 mg/mmol were 1.75 (95% CI 1.12–2.72, *p* = 0.02) for lacunar stroke, 1.51 (95% CI 0.78–2.94, *p* = 0.19) for large artery atherosclerotic stroke and 1.22 (95% CI 0.64–2.3, *p* = 0.52) for cardio-embolic stroke.

**Table 3:** Hazard Ratios and 95% Confidence Intervals for Ischemic Stroke Subtypes by Low-grade Albuminuria Among HUNT 2 Participants, (*n* = 7261)

		Model 1			Model 2		
ACR, mg/mmol	Cases/Person time in years	HR	95% CI	p for trend	HR	95% CI	p for trend
**Lacunar stroke**							
< 1	96/55733	1	(ref)		1	(ref)	
1 - < 2	38/16548	1.19	(0.82–1.74)		1.09	(0.75–1.6)	
2 - < 3	11/3786	1.45	(0.77–2.71)		1.25	(0.66–2.35)	
≥ 3	28/6130	2.25	(1.48–3.45)	< 0.001	1.75	(1.12–2.72)	0.019
**Cardio-embolic stroke**							
< 1	56/55733	1	(ref)		1	(ref)	
1 - < 2	21/16548	0.98	(0.59–1.62)		0.96	(0.58–1.6)	
2 - < 3	7/3786	1.36	(0.62–2.98)		1.23	(0.56–2.73)	
≥ 3	12/6130	1.43	(0.76–2.67)	0.242	1.22	(0.64–2.3)	0.518
**Large artery atherosclerotic stroke**							
< 1	45/55733	1	(ref)		1	(ref)	
1 - < 2	16/16548	1.03	(0.58–1.83)		0.98	(0.55–1.76)	
2 - < 3	7/3786	1.87	(0.84–4.16)		1.49	(0.66–3.35)	
≥ 3	12/6130	2	(1.05–3.79)	0.022	1.51	(0.78–2.94)	0.186
**Stroke of other determined etiology**							
< 1	6/55733	1	(ref)		1	(ref)	
1 - < 2	2/16548	0.9	(0.22–5.52)		1	(0.19–5.13)	
2 - < 3	0/3786						
≥ 3	2/6130	2.4	(0.58–14.64)	0.353	2.09	(0.37–11.72)	0.619
**Stroke of undetermined etiology**							
< 1	183/55733	1	(ref)		1	(ref)	
1 - < 2	85/16548	1.16	(0.9–1.51)		1.1	(0.85–1.43)	
2 - < 3	24/3786	1.38	(0.9–2.12)		1.24	(0.8–1.9)	
≥ 3	52/6130	1.83	(1.34–2.49)	< 0.001	1.53	(1.11–2.11)	0.009

*ACR* Urine albumin-creatinine ratio; *CI* Confidence interval; *HR* Hazard ratio

Model 1 is age adjusted

Model 2 is additionally adjusted for sex, smoking status, educational status, BMI, EGFR, DM, systolic blood pressure, non-HDL Cholesterol, Triglycerides

Sensitivity analysis using multiple imputation for missing covariates yielded nearly identical estimates to those in the main analyses (Additional Tables 1 and 2).

In an additional sensitivity analysis, ischemic stroke was defined by ICD-10 diagnoses in the patient administrative system from HUNT2 examination until September 6th, 2002, and validated ischemic stroke was used afterwards as in our main analysis. We observed slightly lower estimates for the association between albuminuria and risk of ischemic stroke when compared to our main analysis. (Additional Table 3) A further sensitivity analysis, exploring the influence of each modifiable risk factor on the association between albuminuria and risk of stroke and stroke subtypes separately, suggested that blood pressure, diabetes mellitus and triglycerides may play a more important role in the pathophysiology of lacunar stroke than in cardio-embolic stroke. (Additional Table 4).

## Discussion

In this prospective study with a median follow-up time of 15 years, our findings indicate a potential positive association between low-grade albuminuria and risk of ischemic stroke, mainly with moderate albuminuria. Among the various stroke subtypes, lacunar stroke appeared to have the strongest association.

### Comparison with earlier studies

We found our results to be consistent with previous studies that have reported a positive association between albuminuria and risk of ischemic stroke [14, 26–32]. As in some earlier studies, our results suggest a potentially increased risk of ischemic stroke already at a slightly elevated ACR, and especially with moderately increased values [31, 33]. For hemorrhagic strokes, although we found no indication for a dose-response relationship, our estimates for the association between mild albuminuria and the risk of hemorrhagic stroke were of a similar magnitude than those reported in other studies among patients with type 1 diabetes [34], chronic kidney disease [32] and older populations [35]. We were not able to distinguish between hypertensive hemorrhagic stroke and hemorrhagic stroke attributable to cerebral amyloid angiopathy, and the non-linear association between ACR and risk of hemorrhagic stroke may be due to different underlying pathophysiological mechanisms.

Few studies have examined the association of albuminuria with all different stroke sub-types simultaneously, and only one of them had a prospective design [14, 36].

As with the Japanese study, the research conducted by our team, revealed comparable results when assessing the association between albuminuria and the risk of lacunar stroke, showing that the risk was higher than those between albuminuria and other stroke subtypes [14].

Our findings are also supported by a meta-analysis by Georgakis et al. who reported that albuminuria was associated with magnetic resonance imaging markers of CSVD [13]. However, magnetic resonance imaging markers of CSVD, present as lacunes of presumed vascular origin, often correspond to silent brain infarcts that might not be directly comparable with clinical stroke diagnoses that we used in our study [37].

In accordance with the findings of Nakamura et al., we found no evidence for associated risk between albuminuria and cardio-embolic stroke [14]. This was somewhat unexpected given the results of a meta-analysis that reported a positive association between albuminuria and atrial fibrillation, a main risk factor for cardio-embolic stroke [38]. An explanation for the difference between our results and the increased prevalence of albuminuria among patients with atrial fibrillation may be that adequate anticoagulant therapy in these patients reduces their risk of stroke [39]. Atrial fibrillation is accompanied by pathological processes in the myocardium, like myocardial infarction, atrial dilatation or myocardial fibrosis [38].

Lacunar stroke, however, may be more directly associated with endothelial dysfunction, damage of the blood brain barrier, leading to microatheroma, lipohyalinosis and occlusion or thromboembolic ischemic lesion in the small blood vessels of the brain [2, 10, 11]. A probable pathophysiological process leading to endothelial damage has been described by the “strain vessel” hypothesis by Ito et al. [9] According to this hypothesis, vessels in the brain and the kidneys share hemodynamic and anatomical similarities and these strained vessels are recognized to share similar pressure-induced injuries due to increased pulsatile blood pressure amplitudes and stiffness due to atherosclerosis [40]. Therefore, it is possible that changes in endothelial function in the kidneys leading to leakage of albumin to the urine may serve as a marker for generalized endothelial dysfunction. Thus, low-grade albuminuria might indicate both cerebrovascular and renal injury [9]. Other possible causes for endothelial damage include aging, high salt use, chronic inflammation and genetic causes which are described in more detail in a reviews by Wardlaw et al. and Ihara et al. [10, 11]

### Strengths and limitations

Due to the stability of the HUNT study population and the access to free health care to everyone in Norway, our study had a virtually complete follow-up with a median time of 15 years. Measuring of albuminuria relied on the 3 samples of morning urine supplied by our participants, reduces misclassification by artificially increased albumin excretion [41]. Furthermore, our study used validated stroke diagnoses where we were able to distinguish between different subtypes of stroke. In addition, we were able to adjust for a broad range of covariates.

Despite its broad use in previous research, the TOAST classification system is known to classify a high proportion of strokes into the heterogenous group of ‘stroke of undetermined etiology’ [42] which might have biased our results. Nonetheless, it is more important that the groups of lacunar stroke, cardio-embolic stroke and stroke due to large artery arteriosclerosis were well defined. Any misclassification of stroke subtypes as stroke of undetermined etiology would most probably not depend on ACR and thus might have weakened, but not inflated our results.

Notwithstanding this, the inter-rater reliability for ischemic stroke subtypes was moderate and may have led to non-differential misclassification and a possible reduction of the estimates. Furthermore, we could not compare all first ever stroke subtypes with each other, because we identified and categorized only first ever stroke diagnosis.

It should be noted that we lacked information on the specific type of antihypertension medication which may have been important because angiotensin-converting enzyme inhibitors and angiotensin receptor blockers reduce albuminuria [13, 43].

Only strokes diagnosed in a hospital were included, and thus we were unable to factor fatal strokes that occurred outside the hospital as well as non-fatal strokes not admitted to hospital. However, despite this in our inter-rater reliability analysis, our specificity was 100%. We shall emphasize that with perfect specifity, lower sensitivity, in absence of a differential misclassification, does not bias the risk ratios [44].

Participants collected urine samples at home, and we cannot completely rule out the possibility of interchanging urine samples or contamination, although they were orally and written informed how to collect urine and recommended to store the urine samples in a refrigerator until returning to the laboratory. We expect this error to be unrelated to the risk of stroke, and thus such a non-differential misclassification is likely to bias our results only toward the null. We therefore might under-, but not overestimate the effect of albuminuria on stroke risk in our study.

Originally, the Microalbuminuria study investigated whether the presence of microalbuminuria can be used to predict cardiovascular morbidity and mortality in diabetic and hypertensive patients and for comparison also a sample was drawn among those free of these conditions [17]. Due to this selected population our data are not directly generalizable to other settings. Furthermore, participants were almost exclusively of Caucasian origin, therefore our findings in this Norwegian study might not be directly applicable to other populations.

## Conclusions

In conclusion, our findings suggest that low-grade albuminuria is an important risk factor for ischemic stroke especially for the lacunar subtype. Further studies are necessary to confirm that elevated ACR at relatively low values can increase the risk for lacunar stroke or other degenerative or vascular changes in the brain due to CSVD. Measuring albuminuria is cost effective and easily available. This will afford us the opportunity to estimate the risk for endothelial dysfunction and thus further assess the risk for stroke, stroke subtypes and CSVD.

## Additional Files

**Additional file 1: Figure 1**. Flowchart of the study population.

**Additional file 2: Table 1**. Association between ACR and all ischemic and hemorrhagic stroke, using multiple imputation for missing covariates.

**Additional file 3: Table 2**. Associations between ACR and different ischemic stroke subtypes, using multiple imputation for missing covariates

**Additional file 4: Table 3**. Association between ACR and all ischemic stroke, follow up from HUNT2 examination.

**Additional file 5: Table 4**. Influence of each modifiable risk factor on the association between albuminuria and risk of all ischemic stroke, lacunar stroke and cardio-embolic stroke.

## Data Availability

Data from the HUNT study used in research projects is available upon reasonable request to the HUNT data access committee (hunt@medisin.ntnu.no).

## Abbreviations

HUNT: Population-based nord-trøndelag health study
HR: Hazard ratio
ACR: Albumin-to-creatinine-ratio
CSVD: Cerebral small vessel disease
GFR: Glomerular filtration rate
EGFR: Estimated GFR
BMI: Body mass index
BP: Systolic blood pressure
DM: Diabetes mellitus
KDIGO: Kidney disease: improving global outcomes
ICD-10: International classification of diseases-tenth revision
TOAST: Acute stroke treatment classification system
SD: Standard deviation
IQR: Interquartile range
CI: Confidence interval
