# Supplementary figures and images for: Low grade albuminuria as a risk factor for subtypes of stroke - the HUNT Study in Norway
Source: BMC Neurol. 2020 May 2;20:170. doi: 10.1186/s12883-020-01746-9 (PMC7196218; doi:10.1186/s12883-020-01746-9)

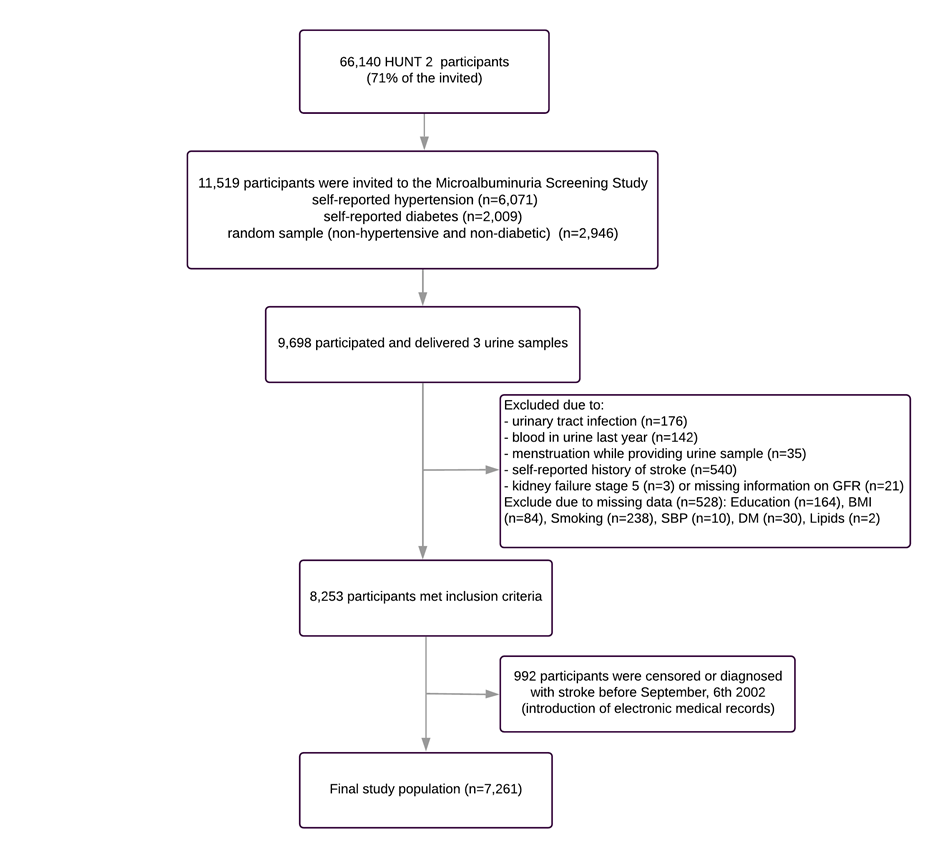


Additional Figure I. Flowchart of the study population

Supplement: Supplementary file 1 — Additional file 1: Figure 1. Flowchart of the study population. [file 12883_2020_1746_MOESM1_ESM.docx]
