# Supplementary material for: Low grade albuminuria as a risk factor for subtypes of stroke - the HUNT Study in Norway
Source: BMC Neurol. 2020 May 2;20:170. doi: 10.1186/s12883-020-01746-9 (PMC7196218; doi:10.1186/s12883-020-01746-9)
Supplement: Supplementary file 2 — Additional file 2: Table 1. Association between ACR and all ischemic and hemorrhagic stroke, using multiple imputation for missing covariates. [file 12883_2020_1746_MOESM2_ESM.docx]

| **Additional Table I. Hazard Ratios and 95% Confidence Intervals for all Ischemic Strokes and Hemorrhagic Stroke Among HUNT 2 Participants, Multiple Imputation (n=7661)** | | | | | | | | | | |
| --- | --- | --- | --- | --- | --- | --- | --- | --- | --- | --- |
| ACR | Cases/Person time in years | | Model 1 | | | | Model 2 | | | |
| mg/mmol |  |  | HR | 95% CI | p for trend | | HR | 95% CI | p for trend | |
| **All ischemic stroke** | | |  |  |  |  |  |  |  |  |
| <1 | 410/57693 |  | 1 | (ref) |  |  | 1 | (ref) |  | |
| 1 - < 2 | 173/17463 |  | 1.11 | (0.93-1.33) |  |  | 1.05 | (0.87-1.25) |  | |
| 2 - < 3 | 50/4048 |  | 1.32 | (0.98-1.77) |  |  | 1.16 | (0.86-1.57) |  | |
| ≥3 | 115/6499 |  | 1.89 | (1.54-2.33) | <0.001 |  | 1.56 | (1.25-1.93) | <0.001 | |
| **Hemorrhagic stroke** | | | | | | | | | | |
| <1 | 30/57693 |  | 1 | (ref) |  |  | 1 | (ref) |  | |
| 1 - < 2 | 31/17463 |  | 2.47 | (1.49-4.08) |  |  | 2.49 | (1.49-4.15) |  |  |
| 2 - < 3 | 4/4048 |  | 1.33 | (0.47-3.77) |  |  | 1.29 | (0.45-3.7) |  |  |
| ≥3 | 9/6499 |  | 1.84 | (0.87-3.88) | 0.042 |  | 1.77 | (0.82-3.81) | 0.062 | |
| Abbreviations: ACR, urine albumin-creatinine ratio; CI, confidence interval; HR, hazard ratio.  Model 1 is age adjusted.  Model 2 is additionally adjusted for sex, smoking status, educational status, BMI, EGFR, DM, systolic blood pressure, non-HDL Cholesterol, Triglycerides | | | | | | | | | | |
