# Supplementary material for: Low grade albuminuria as a risk factor for subtypes of stroke - the HUNT Study in Norway
Source: BMC Neurol. 2020 May 2;20:170. doi: 10.1186/s12883-020-01746-9 (PMC7196218; doi:10.1186/s12883-020-01746-9)
Supplement: Supplementary file 3 — Additional file 3: Table 2. Associations between ACR and different ischemic stroke subtypes, using multiple imputation for missing covariates [file 12883_2020_1746_MOESM3_ESM.docx]

| **Additional Table II. Hazard Ratios and 95% Confidence Intervals for Stroke Subtype by Albuminuria Among HUNT 2 Participants. Multiple Imputation (n=7661)** | | | | | | | | | |  |  |
| --- | --- | --- | --- | --- | --- | --- | --- | --- | --- | --- | --- |
| ACR | Cases/Person time in years | | Model 1 | | | | Model 2 | | | | |
| mg/mmol |  |  | HR | 95% CI | p for trend | | HR | 95% CI | p for trend | | |
| **Lacunar stroke** | | | | | | | | | | | |
| <1 | 100/57693 |  | 1 | (ref) |  |  | 1 | (ref) |  | | |
| 1 - < 2 | 39/17463 |  | 1.15 | (0.79-1.67) |  |  | 1.06 | (0.73-1.55) |  | | |
| 2 - < 3 | 12/4048 |  | 1.45 | (0.8-2.65) |  |  | 1.26 | (0.69-2.32) |  | | |
| ≥3 | 29/6499 |  | 2.19 | (1.44-3.31) | <0.001 |  | 1.7 | (1.1-2.63) | 0.022 | | |
| **Cardio embolic stroke** | | | | | | | | | | | |
| <1 | 59/57693 |  | 1 | (ref) |  |  | 1 | (ref) |  | | |
| 1 - < 2 | 21/17463 |  | 0.91 | (0.55-1.51) |  |  | 0.89 | (0.54-1.48) |  | |  |
| 2 - < 3 | 7/4048 |  | 1.24 | (0.56-2.72) |  |  | 1.12 | (0.51-2.48) |  | |  |
| ≥3 | 13/6499 |  | 1.44 | (0.79-2.63) | 0.271 |  | 1.22 | (0.65-2.48) | 0.585 | | |
| **Large artery atherosclerotic stroke** | | | | | | | | | | | |
| <1 | 48/57693 |  | 1 | (ref) |  |  | 1 | (ref) |  | | |
| 1 - < 2 | 17/17463 |  | 1.0 | (0.57-1.74) |  |  | 0.94 | (0.54-1.65) |  | |  |
| 2 - < 3 | 7/4048 |  | 1.68 | (0.76-3.71) |  |  | 1.35 | (0.6-3.02) |  | |  |
| ≥3 | 13/6499 |  | 1.96 | (1.06-3.64) | 0.028 |  | 1.49 | (0.78-2.83) | 0.218 | | |
| **Stroke of other determined etiology** | | | | | | | | | | | |
| <1 | 6/57693 |  | 1 | (ref) |  |  | 1 | (ref) |  | | |
| 1 - < 2 | 2/17463 |  | 1.08 | (0.22-5.42) |  |  | 1.04 | (0.19-5.1) |  | |  |
| 2 - < 3 | 0/4048 |  |  |  |  |  |  |  |  | |  |
| ≥3 | 2/6499 |  | 2.84 | (0.57-14.23) 0.370 | | | 2.06 | (0.37-11.6) 0.625 | | | |
| **Stroke of undetermined etiology** | | | | | | | | | | | |
| <1 | 197/57693 |  | 1 | (ref) |  |  | 1 | (ref) |  | | |
| 1 - < 2 | 94/17463 |  | 1.18 | (0.92-1.51) |  |  | 1.11 | (0.87-1.42) |  | |  |
| 2 - < 3 | 24/4048 |  | 1.24 | (0.81-1.89) |  |  | 1.12 | (0.73-1.71) |  | |  |
| ≥3 | 58/6499 |  | 1.86 | (1.39-2.5) | <0.001 |  | 1.57 | (1.16-2.14) | 0.007 | | |
| Abbreviations: ACR, urine albumin-creatinine ratio; CI, confidence interval; HR, hazard ratio.  Model 1 is age adjusted.  Model 2 is additionally adjusted for sex, smoking status, educational status, BMI, EGFR, DM, systolic blood pressure, non-HDL Cholesterol, Triglycerides | | | | | | | | | | | |
