# Supplementary material for: Low grade albuminuria as a risk factor for subtypes of stroke - the HUNT Study in Norway
Source: BMC Neurol. 2020 May 2;20:170. doi: 10.1186/s12883-020-01746-9 (PMC7196218; doi:10.1186/s12883-020-01746-9)
Supplement: Supplementary file 4 — Additional file 4: Table 3. Association between ACR and all ischemic stroke, follow up from HUNT2 examination. [file 12883_2020_1746_MOESM4_ESM.docx]

| **Additional Table III. Hazard Ratios and 95% Confidence Intervals for all Ischemic Stroke* by Albuminuria With a Follow up from HUNT2 Examination (n=8255)** | | | | |
| --- | --- | --- | --- | --- |
| ACR  mg/mmol | Cases/Person time in years | HR | 95% CI | p for trend |
| <1 | 490/84850 | 1 | (ref) |  |
| 1 -< 2 | 222/27333 | 0.99 | (0.84-1.16) |  |
| 2 -< 3 | 67/6610 | 1.1 | (0.85-1.43) |  |
| ≥ 3 | 169/11584 | 1.44 | (1.2-1.73) | <0.001 |
| Abbreviations: ACR, urine albumin-creatinine ratio; CI, confidence interval; HR, hazard ratio.  Model is adjusted for age, sex, smoking status, educational status, BMI, EGFR, DM, systolic blood pressure, non-HDL Cholesterol, Triglycerides.  * includes unvalidated ischemic stroke diagnosis (ICD-10 code I63 from the patient administrative system) until 2002 | | | | |
