# Supplementary material for: Low grade albuminuria as a risk factor for subtypes of stroke - the HUNT Study in Norway
Source: BMC Neurol. 2020 May 2;20:170. doi: 10.1186/s12883-020-01746-9 (PMC7196218; doi:10.1186/s12883-020-01746-9)
Supplement: Supplementary file 5 — Additional file 5: Table 4. Influence of each modifiable risk factor on the association between albuminuria and risk of all ischemic stroke, lacunar stroke and cardio-embolic stroke. [file 12883_2020_1746_MOESM5_ESM.docx]

| **Additional Table IV.** Hazard Ratios and 95% Confidence Intervals for all Ischemic Stroke, Lacunar Stroke and cardio embolic Stroke with and without modifiable risk factors as co-factors in the fully adjusted model. | | | | | | | | | | | |
| --- | --- | --- | --- | --- | --- | --- | --- | --- | --- | --- | --- |
|  | | ACR |  | All Ischemic Stroke | |  | Lacunar Stroke | |  | Cardio embolic Stroke | |
|  | mg/mmol | | | HR | 95% CI |  | HR | 95% CI |  | HR | 95% CI |
| Fully adjusted model | | <1 |  | 1 | Ref. |  | 1 | Ref. |  | 1 | Ref. |
|  | | 1-<2 |  | 1.06 | 0.88-1.26 |  | 1.09 | 0.75-1.60 |  | 0.96 | 0.58-1.60 |
|  | | 2-<3 |  | 1.25 | 0.92-1.69 |  | 1.25 | 0.66-2.35 |  | 1.23 | 0.56-2.73 |
|  | | >=3 |  | 1.56 | 1.24-1.95 |  | 1.75 | 1.12-2.72 |  | 1.22 | 0.64-2.33 |
| Fully adjusted model HT instead of SBP | | <1 |  | 1 | Ref. |  | 1 | Ref. |  | 1 | Ref. |
|  | | 1-<2 |  | 1.09 | 0.90-1.31 |  | 1.12 | 0.76-1.64 |  | 0.99 | 0.60-1.64 |
|  | | 2-<3 |  | 1.30 | 0.96-1.75 |  | 1.29 | 0.69-2.42 |  | 1.28 | 0.58-2.83 |
|  | | >=3 |  | 1.64 | 1.32-2.05 |  | 1.83 | 1.18-2.83 |  | 1.29 | 0.68-2.44 |
| Fully adjusted Model without SBT or HT | | <1 |  | 1 | Ref. |  | 1 | Ref. |  | 1 | Ref. |
|  | | 1-<2 |  | 1.11 | 0.92-1.34 |  | 1.14 | 0.78-1.67 |  | 1.00 | 0.61-1.67 |
|  | | 2-<3 |  | 1.34 | 0.99-1.80 |  | 1.32 | 0.70-2.48 |  | 1.31 | 0.59-2.88 |
|  | | >=3 |  | 1.68 | 1.35-2.10 |  | 1.87 | 1.21-2.90 |  | 1.31 | 0.69-2.48 |
| Fully adjusted Model without DM | | <1 |  | 1 | Ref. |  | 1 | Ref. |  | 1 | Ref. |
|  | | 1-<2 |  | 1.08 | 0.90-1.30 |  | 1.12 | 0.77-1.65 |  | 0.97 | 0.58-1.62 |
|  | | 2-<3 |  | 1.28 | 0.95-1.73 |  | 1.31 | 0.70-2.46 |  | 1.25 | 0.56-2.77 |
|  | | >=3 |  | 1.64 | 1.32-2.05 |  | 1.91 | 1.23-2.96 |  | 1.25 | 0.66-2.38 |
| Fully adjusted Model without BMI | | <1 |  | 1 | Ref. |  | 1 | Ref. |  | 1 | Ref. |
|  | | 1-<2 |  | 1.06 | 0.88-1.28 |  | 1.09 | 0.74-1.60 |  | 0.96 | 0.57-1.59 |
|  | | 2-<3 |  | 1.26 | 0.93-1.71 |  | 1.27 | 0.67-2.39 |  | 1.26 | 0.57-2.78 |
|  | | >=3 |  | 1.57 | 1.25-1.96 |  | 1.78 | 1.14-2.77 |  | 1.24 | 0.65-2.38 |
| Fully adjusted Model without non-HDL | | <1 |  | 1 | Ref. |  | 1 | Ref. |  | 1 | Ref. |
|  | | 1-<2 |  | 1.06 | 0.88-1.28 |  | 1.10 | 0.75-1.61 |  | 0.96 | 0.58-1.61 |
|  | | 2-<3 |  | 1.25 | 0.93-1.69 |  | 1.24 | 0.66-2.33 |  | 1.23 | 0.56-2.73 |
|  | | >=3 |  | 1.56 | 1.24-1.95 |  | 1.75 | 1.12-2.73 |  | 1.22 | 0.64-2.34 |
| Fully adjusted Model without TG | | <1 |  | 1 | Ref. |  | 1 | Ref. |  | 1 | Ref. |
|  | | 1-<2 |  | 1.06 | 0.88-1.28 |  | 1.12 | 0.76-1.64 |  | 0.96 | 0.58-1.60 |
|  | | 2-<3 |  | 1.25 | 0.92-1.69 |  | 1.25 | 0.66-2.36 |  | 1.23 | 0.56-2.73 |
|  | | >=3 |  | 1.56 | 1.24-1.95 |  | 1.82 | 1.17-2.84 |  | 1.21 | 0.63-2.31 |
| Fully adjusted Model without Smoking | | <1 |  | 1 | Ref. |  | 1 | Ref. |  | 1 | Ref. |
|  | | 1-<2 |  | 1.06 | 0.88-1.28 |  | 1.09 | 0.74-1.50 |  | 0.96 | 0.57-1.59 |
|  | | 2-<3 |  | 1.26 | 0.93-1.70 |  | 1.23 | 0.65-2.31 |  | 1.21 | 0.55-2.68 |
|  | | >=3 |  | 1.57 | 1.25-1.96 |  | 1.70 | 1.09-2.65 |  | 1.20 | 0.63-2.28 |
| Fully adjusted Model is adjusted for age, sex, smoking status, educational status, Body Mass Index (BMI), Estimated Glomerular Filtration Ratio (EGFR), Diabetes mellitus (DM), systolic blood pressure (SBP), non-HDL Cholesterol and Triglycerides (TG). ACR: Albumin to Creatinine Ratio; HR: Hazard Ratio; CI: Confidence Interval; HT: Hypertension defined by Systolic blood pressure >140mmHg or use of blood pressure medication. | | | | | | | | | | | |
